# Effects of a Traditional Caraway Formulation on Experimental Models of Vitiligo and Mechanisms of Melanogenesis

**DOI:** 10.1155/2021/6675657

**Published:** 2021-04-19

**Authors:** Abudujilili Abuduaini, Xueying Lu, Deng Zang, Tao Wu, Haji Akbar Aisa

**Affiliations:** ^1^State Key Laboratory Basis of Xinjiang Indigenous Medicinal Plants Resource Utilization, and Key Laboratory of Plant Resources and Chemistry in Arid Regions, Xinjiang Technical Institute of Physics and Chemistry, Chinese Academy of Sciences, Urumqi 830011, China; ^2^University of Chinese Academy of Sciences, Beijing 100039, China

## Abstract

**Background:**

Kursi Karwiya or caraway tablet (CWT), a traditional medicine formula, is widely used in Xinjiang, China, for treating vitiligo, a common autoimmune disease for which there is currently no satisfactory cure. Clinical interventions include pharmacological treatment with psoralens, often in conjunction with UVA radiation, but toxic side effects limit this application. Studies on the activities and mechanisms of CWT are scarce.

**Objective:**

To investigate the in vitro and in vivo effects of CWT in B16 cell line and in animal models of vitiligo, further exploring its mechanisms of regulating melanogenesis.

**Methods:**

Effects of CWT on melanin synthesis in B16 cells and mushroom tyrosinase activity were investigated in vitro. The signaling pathway of melanogenesis in murine B16 melanoma cells was examined by Western blotting. Two different animal models were used, vitiligo induced by hydroquinone in the mouse model and by hydrogen peroxide in the guinea pig model. Relevant biochemical parameters in blood and skin tissue were measured, and visual inspection, histopathology, and immunohistochemical analysis of treated areas were carried out.

**Results:**

CWT produced changes in biochemical parameters including TYR, MDA, MAO, AChE, IL-6, INF-*α*, *β*-EP, and cAMP in blood and/or skin tissue and in regulating melanogenesis. After treatment with CTW, skin color, melanin containing hair follicles, and expression of TYR, TRP-1, and TRP-2 in the skin of animals were significantly affected.

**Conclusions:**

CWT alleviated many of detrimental effects in both models of vitiligo. Tyrosinase activity and melanin content in B16 cells were increased, at least in part, via activation of the PKA p38 MAPK signaling pathways. Our results show that CWT produces beneficial effects on parameters of vitiligo and is worthy of further investigation for use in this distressing autoimmune disorder which currently has no effective cure.

## 1. Introduction

Vitiligo is an autoimmune cutaneous disease of pigmentation, characterized by the development of well-defined white patches on the skin and mucous membranes [1]. Currently, there is no satisfactory cure for this distressing disorder.

Vitiligo is characterised by the destruction of melanocytes and inhibition of the melanin synthesis pathway [2]. Melanocytes from the skin of vitiligo patients, compared to normal melanocytes [3, 4], die more readily in response to exogenous oxidative stressors, with elevated levels of reactive oxygen species [5, 6] and greater endoplasmic reticulum stress. These effects may be replicated by exposing the skin to phenols such as 4-tert-butylphenol and hydroquinone monobenzyl ether (i.e., monobenzone), which worsen clinical depigmentation in vitiligo patients [7].

Treatments for vitiligo include pharmacological and surgical interventions, phototherapy, and cosmetic measures. Most are costly and some are painful, and none is completely effective. The use of psoralens with UVA radiation (P-UVA) is undoubtedly effective but associated with a higher risk of skin cancer [8]. Psoralens have significant side effects, which include hepatotoxicity, nausea and vomiting, and photosensitivity [9], and their effects are cumulative [10].

Herbal medicine is commonly used in the treatment of vitiligo and as adjunctive for other treatments and provides the potential for wider use if safety and efficacy can be demonstrated. Many herbal formulae for vitiligo include psoralen-containing herbs, in combination with other plant extracts, including CWT. The presence of other herbs is intended to provide a multitargeted approach to treatment, enhance efficacy, and mitigate side effects of other ingredients, which is part of traditional medicine theory. We therefore investigated the effects of CWT in several pharmacological models and compared them to baidianfeng capsule (BC or vitiligo capsule), a patent TCM (traditional Chinese medicine) formula, used throughout China to treat vitiligo. The main active constituents of BC are psoralens, but this formula also contains other herbs.

“Kursi Karwiya” or “Kursi Carwiya” (caraway tablet, CWT) is a traditional herbal formula used in Western China for the treatment of vitiligo [11]. CWT contains caraway, *Carum carvi* L, as the main constituent, with *Psoralea corylifolia* L., *Tribulus terrestris* L., *Trachyspermum ammi* (L.) Sprague, and *Operculina turpethum* L. in the ratio 2 : 1 : 1 : 1 : 1. The composition of CWT is based on traditional medicine principles, and the constituent herbs are well-known medicinal plants and have been chemically and pharmacologically investigated to some extent. Some of these studies provide limited evidence for their inclusion in a formula for vitiligo.

Caraway, *Carum carvi*, seed (fruit) is the major ingredient of CWT. Caraway is used as a food all over the world and plays a part in many systems of traditional medicine. It contains an essential oil composed mainly of carvone, up to 60%, and has carminative, anti-inflammatory, antioxidant, and antimicrobial effects [12]. The use of caraway in skin disorders is not well-documented, but a recent study by Kang et al. [13] suggests that carvone has the potential to be a useful treatment in melanomas and related disorders. The antioxidant effects of caraway may be protective of melanocytes, and the carminative effect would counteract the griping effect of other purgative ingredients.

*Psoralea corylifolia* seed is used in Chinese and Ayurvedic medicine to promote skin pigmentation. It contains psoralen and isopsoralen, which are known treatments for vitiligo. The seeds also contain chalcones, isoflavones such as corylinin and daidzein, and bakuchiol, a monoterpene phenol and its dimers [14]. This is the only component of CWT containing psoralens, and as known active constituents, they were measured.

*Trachyspermum ammi*, ajowan seed, is used in food and in Ayurvedic and other systems of medicine as an antispasmodic and carminative. It contains an essential oil, the main component of which is thymol (∼60%), with p-cymene (∼15%), *γ*-terpinene, and others. It is not well-documented for use in skin disease, except as a topical antiseptic. However, a study measuring its effects on skin thickness in rats confirmed immunomodulatory and immunostimulant activities in skin tissues [15].

*Tribulus terrestris* fruits (TTF) contain saponins, the terrestrosins, which are based on protodioscin, phytosterols including *β*-sitosterol and stigmasterol, and flavonoids. Extracts are used traditionally to treat inflammatory and allergic skin diseases, including atopic dermatitis (AD). AD has a complex aetiology involving activation of immunological and inflammatory pathways. Kang et al. [16] found that *Tribulus* fruit extract inhibited skin inflammation in AD by blocking infiltration of inflammatory cells such as T cells and eosinophils. These properties may be relevant for the inclusion of TTF in CWT for use in vitiligo.

*Operculina turpethum*, black turpeth root, has a long history of medicinal use for a variety of disorders, including skin disorders and specifically vitiligo, where it is an important herb in the Indian system of medicine, Ayurveda [17]. It contains resinous glycosides including *α*- and *β*-turpethein, turpethenic acids, coumarins such as scopoletin, phytosterols including *β*-sitosterol, lupeol, scopoletin, betulin, and salicylic acid, and a series of dammarane saponins known as operculinosides. Extracts have been shown to have anti-inflammatory and antibacterial properties and the saponins hepatoprotective effects [17]. Salicylic acid is a keratolytic widely used in skin disorders. The resins are strongly purgative in high doses.

The scientific rationale for this formula is not proven, although the presence of *Psoralea* is to be expected. However, *Psoralea* is a minor part of the formula, and no other constituent herb contains psoralens. Therefore, the effects of CWT are likely to be due to multiple mechanisms, including those induced by psoralens, and potentially also via antioxidant and immunological mechanisms.

Despite its long history of use, the pharmacological mechanisms behind the effects of CWT have not been elucidated. We have investigated these in validated mouse and guinea pig models of vitiligo, induced by hydroquinone and hydrogen peroxide, respectively, and its mechanisms in regulating melanogenesis and involvement of PKA signaling. A schematic graph showing the design and the core findings of the study can be seen in Supplementary Materials.

## 2. Materials and Methods

### 2.1. Sample Preparation

#### 2.1.1. Preparation of Caraway Tablet

*Carum carvi, Psoralea corylifolia, Tribulus terrestris*, and *Trachyspermum ammi* seeds and *Operculina turpethum* root were identified, and voucher specimens were kept at the Xinjiang Technical Institute of Physics and Chemistry, Urumqi. The preparation of extract of CWT was based on previous methods (Patent No. CN 107569532 A). The protocol for preparation of caraway tablet is given in Supplementary Materials.

#### 2.1.2. Quantification of Psoralen and Isopsoralen in CWT

CWT was dissolved in methanol and after ultrasonic extraction, filtered and analyzed for psoralen and isopsoralen, using HPLC (high-performance liquid chromatography, SHIMADZU, Japan) and a mobile phase of methanol and water (55 : 45, v/v) for 1 mL min^−1^ at 30°C and UV detection at 245 nm. Three samples were analyzed and found to contain an average of 2.21 mg/g psoralen and 1.54 mg/g isopsoralen.

#### 2.1.3. Preparation of Blood and Skin Samples

After collection of blood samples, sera were isolated by centrifugation and stored at −70°C.

Skin tissue samples were homogenized and mixed in an oscillator at 4°C for 60 min before being centrifuged at 4°C for 15 min, and the supernatant was collected.

### 2.2. Animals and Treatment

#### 2.2.1. Animals

Male and female (50 : 50) were obtained from Xinjiang Medical University and kept at 18–25°C and 40–60% humidity, with food and water ad libitum. Briefly, they were randomly divided into six groups: normal control (NC, untreated), model group (disease control), positive controls (BC group), and low-, medium-, and high-dose CWT (CWT-L, CWT-M, and CWT-H) groups, respectively (Tables 1-2). Hairs were shaved from the dorsal skin and smeared with distilled water for 50 days (normal control group) with administration of purified water orally for 30 days; all other groups were smeared with hydroquinone or hydrogen peroxide (5%) for 50 days, with administration of purified water and either BC or CWT orally for 30 days, starting at day 21. Animals were sacrificed 12 h after the final administration, and blood and skin tissue were harvested.

#### 2.2.2. Dose Selection

The clinical dosage of CWT crude extract is 0.9 g/person/day, which equates in mice to approximately 200 mg/kg and in guinea pig to ∼80 mg/kg [18]. The low dose used in our experiments was half of this and the high dose twice of this and approximates to a dose of psoralen of 0.44 mg/kg in CWT-M (200 mg/kg) and 0.88 mg/kg in CWT-H (400 mg/kg).

The clinical dosage of BC is 3.6 g/person/day, which equates to 720 mg/kg in mice and 270 mg/kg in guinea pig, containing doses of psoralen of approximately 1.33 and 0.5 mg/kg.

#### 2.2.3. Measurement of Melanin-Containing Hair Follicles

The melanin-containing hair follicles were observed by conventional histological analysis [14]. A portion of the skin was cut and fixed with 10% neutral-buffered formalin, followed by paraffin embedding. Hematoxylin and eosin staining was performed on sections of approximately 5 mm, fifty hair follicles were observed under the light microscope (×100) (Olympus Optical Co, Ltd, Tokyo, Japan), and melanin-containing hair follicles were counted.

#### 2.2.4. Biochemical Measurements

Malondialdehyde (MDA), monoamine oxidase (MAO), and acetylcholinesterase (AChE) in skin tissue were determined using commercial kits (Nanjing Jiancheng Bioengineering Co. Ltd., China). Absorbance of AChE was measured at 520 nm, MAO at 242 nm, and MDA at 532 nm, respectively, using a microplate reader (Multiskan Go 1510, USA).

#### 2.2.5. Measurement of IL-6, IFN-*γ*, TYR, cAMP, and *β*-EP

The concentrations of cytokines, namely, interferon-gamma (IFN-*γ*), interleukin- (IL-) 6, tyrosinase (TYR), cyclic adenosine monophosphate (cAMP), and *β*-endorphin (*β*-EP) were determined quantitatively in collected serum and tissue samples by enzyme-linked immunosorbent assay (ELISA, Nanjing Jiancheng Bioengineering Co. Ltd., China).

#### 2.2.6. Histopathological Examinations

Skin sections were prepared as described above and stained by hematoxylin and eosin (H&E), or L-DOPA staining was performed and examined under a light microscope.

#### 2.2.7. Immunohistochemistry Analysis

The expression of TYR, TRP-1 (tyrosinase-related protein 1), and TRP-2 (tyrosinase-related protein 2) in the shaved areas of the skin [19, 20] was determined by staining with monoclonal antibodies for tyrosinase, TRP-1, and TRP-2, and the protein expression was determined using Image-Pro Plus 6.0 (Media Cybernetics, Inc., USA).

### 2.3. Cell Culture

The murine B16 melanoma cell line was purchased from the Chinese Academy of Sciences (Shanghai, China). The cells were cultured in HG-DMEM (Gibco) and supplemented with 10% FBS (BI, Biological Industries), 100 *µ*g/mL streptomycin, and 100 U/mL penicillin (Gibco), in a humidified incubator with 5% CO_2_ at 37°C and subcultured every 2 days to maintain logarithmic growth.

### 2.4. Cell Viability Assay

Cell viability was determined using the MTT assay. B16 cells were plated in 96-well dishes at a density of 5 × 10^3^ cells per well. After 24 h, different concentrations of CWT extract were added and incubated for 48 h. 10 *µ*L of MTT (5 mg/ml in PBS) solution was added to each well and incubated at 37°C for another 4 h. Following medium removal, 150 *µ*L of DMSO (dimethyl sulfoxide) was added to each well and gently shaken for 10 min. Optical absorbance was determined at 570 nm with a Spectra Max M5 (Molecular Devices, USA). Absorbance of cells without treatment was regarded as 100% cell survival. Each treatment was performed in quintuplicate, and each experiment was repeated three times.

### 2.5. Melanin Measurement

B16 cells were seeded in a 6-well plate at a density of 1.8 × 10^5^ cells/well. After 24 h of incubation, cells were treated for 48 h with either CWT, 8-MOP (8-methoxypsoralen) 50 *µ*M (positive control), and a vehicle control group with 2 *µ*L DMSO. Cells were washed twice with PBS (PH 7.4), lysed in 100 *µ*L RIPA buffer for 40 min at 4°C, and centrifuged at 12,000 g for 20 min at 4°C. The protein content of the supernatants was measured using the BCA assay, and the pellets were mixed with 190 *µ*L of 1 M NaOH (with 10% DMSO) for 2 h at 60°C. The melanin content was detected at 405 nm by a multiplate reader (Spectra Max M5/M5e) and corrected for the concentrations of proteins.

### 2.6. Tyrosinase Activity

B16 cells were seeded in a 6-well plate at a density of 2.5 × 10^5^ cells/well. After 24 h, they were treated with CWT for 24 h, using 8-MOP 50 *µ*M as a positive control and a vehicle control group of 2 *µ*L DMSO. Cells were lysed with PBS containing 1% Triton X-100 and 1% sodium deoxycholate for 30 min at −20°C and centrifugated at 12,000  × g for 15 min. 90 *µ*L of cell lysate and 10 *µ*L of 10 mM L-DOPA were added to each well, following a 20∼60 min incubation at 37°C in the dark, and the dopachrome was detected at 490 nm. The tyrosinase activity of each sample was calculated and corrected for the concentrations of proteins.

### 2.7. Western Blot Analysis

Protein samples from the melanin content assay were used for Western blot analysis. The lysates were denatured in SDS-PAGE protein loading buffer 5x (Wuhan Boster Biological Technology, Ltd.) separated on 10% SDS-PAGE at 80 V and transferred onto polyvinylidene fluoride membranes for 2 h at 400 A. Membrane blocking was performed with 5% skim milk dissolved in TBS with 1% Tween-20 (TBST) at room temperature for 1 h and the membrane incubated with primary antibodies at dilutions of 1 : 1,000 at 4°C overnight. After washing in TBST, the membranes were incubated with horseradish peroxidase-conjugated secondary antibodies at a dilution of 1 : 2,000 for 1 h and washed again with TBST. Proteins were visualized by enhanced chemiluminescent Western blotting detection reagents (GE Healthcare, Chicago, IL, USA). Densitometric analysis was performed using Quantity One version 3 (BioRad Laboratories, Inc.).

B16 cells were preincubated with H89 (10 *µ*M) for 2 h prior to the addition of CWT (100 *µ*g/mL) and then incubated for 48 h for the measurement of melanin content or incubated for 24 h for the measurement of TYR activity. B16 cells were treated with 0.1% dimethyl sulfoxide and 8-MOP as positive controls or CWT at 5, 50, and 100 *µ*M for 12 h, and cAMP content was measured with a cAMP-ELISA kit. B16 cells were treated with 5, 50, and 100 *µ*M CWT for 48 h, and levels of total and phosphorylated CREB were measured by Western blot. B16 cells were preincubated with H89 (10 *µ*M) for 2 h prior to addition of CWT extract (100 *µ*g/mL) and incubation for 48 h. Phosphorylated CREB and MITF expression levels and levels of CREB induced by PKA, which may activate MITF transcription levels, were investigated using Western blot analysis.

### 2.8. Determination of Intracellular cAMP Levels

B16 melanoma cells were treated with CWT crude extract at 0, 5, 50, or 100 *µ*M at 37°C for 12 h. Intracellular cAMP levels were measured using a cAMP ELISA kit (Cell Biolabs, Inc., San Diego, CA, USA).

### 2.9. Statistical Analysis

The total sample number of 72 for mice and 96 for guinea pigs were selected and randomly divided into six groups for the statistical test. Multiple parameters for the mice and guinea pig were statistically studied. All data were expressed as mean ± SEM. Statistical analysis was performed with one-way ANOVA followed by Tukey's post hoc test for multiple comparison tests. Meanwhile, we used descriptive statistics to obtain mean values and standard deviation (SD). Majority number of significant differences were accepted when *P* < 0.05, and in some cases, the differences were accepted when *P* < 0.01 or *P* < 0.001. The repeated measure statistical test was used for the related measures in vitro experiments. All the results were described using tables or graphs in the results section.

## 3. Results

### 3.1. Color of Hair Regrowth

**Figure 1:**
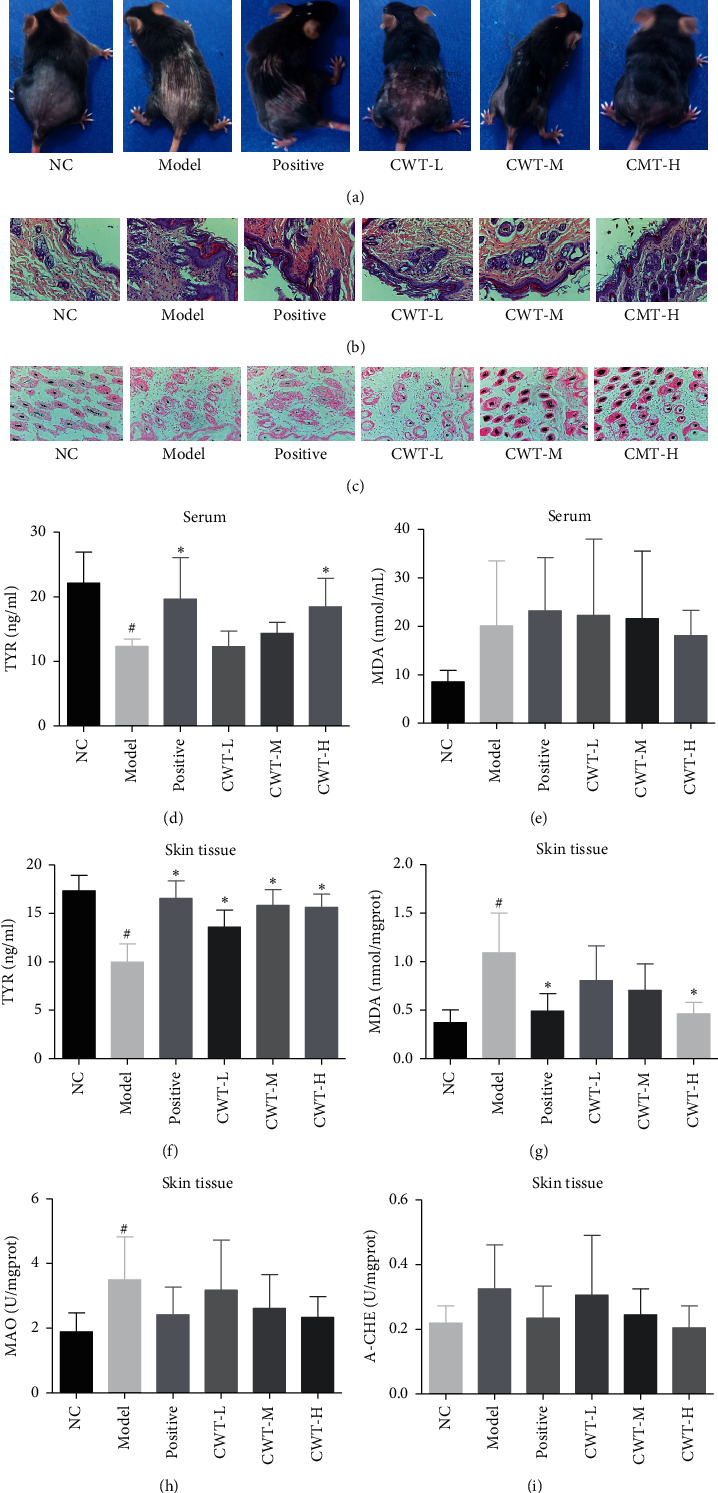
Effect of CWT on the hydroquinone-induced vitiligo mouse model. (a) Color of hair growth; (b) morphology of the skin tissue (H&E staining); (c) number of melanin-containing hair follicles (Lillie staining); (d)-(e) TYR and MDA in serum of mice; and (f)–(i) contents of TYR, MDA, MAO, and AChE in skin tissues. ^#^*P* < 0.05 vs. the normal control group. ^*∗*^*P* < 0.05 vs. the model group. NC, blank control; positive, positive control.

The hair color of mice in all treatment groups turned darker than that of the untreated disease model group. The observed changes were greater in the CWT-M (200 mg/kg) and CWT-H (400 mg/kg) groups and the BC positive control group (Figure 1(a)).

### 3.2. Histological Analysis of Mice Skin

Figure 1(b) shows the effects of CWT on the numbers of melanin-containing follicles, basal melanocytes, and melanin-containing epidermal cells in the shaved skin areas of mice with hydroquinone-induced vitiligo. The disease model group showed a significant decrease compared with the normal control group, and most follicles contained almost no melanin. The number of melanin containing hair follicles in treated areas was increased at all dosages of CWT and in the BC positive control group (*P* < 0.05). Basal melanocyte and melanin-containing epidermal cells were increased significantly (*P* < 0.05) 1 in mice administered 200 and 400 mg/kg CWT (Figures 1(b) and 1(c), Table 3).

### 3.3. The Effect of CWT on TYR, MDA, MAO, and AChE in Serum

The concentration of TYR in serum of the disease model group was significantly lower than that of the normal control (*P* < 0.05) (Figure 1(d)), and the MDA content increased. TYR levels in the BC and the CWT-H groups were significantly higher than the model group (*P* < 0.05), but no significant changes in MDA levels of those groups were observed (*P* < 0.05) (Figure 1(e)).

**Figure 2:**
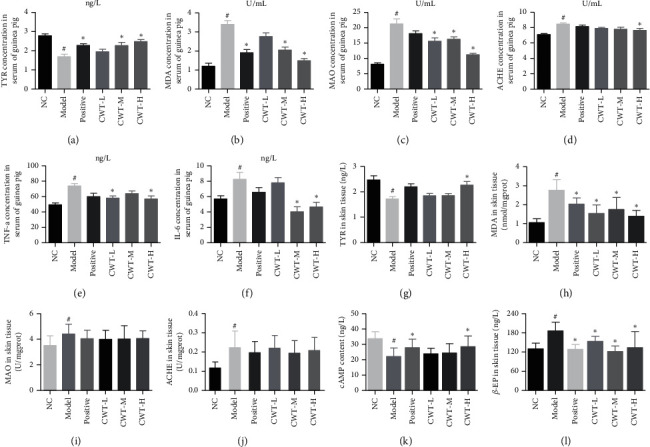
The effect of CWT on blood and skin parameters in guinea pig. Effects on (a) TYR, (b) MDA, (c) MAO, (d) AChE, (e) TNF-*α*, and (f) IL-6 in blood of guinea pig. Effects on contents of (g) TYR, (h) MDA, (i) MAO, (j) AChE, (k) cAMP, and (l) *β*-EP in guinea pig skin. ^#^#*P* < 0.05 vs. the blank control group. ^∗^*∗P* < 0.05 vs. the model group. NC, blank control group; positive, positive control.

The biochemical indicators TYR, MDA, MAO, and AChE were measured in guinea pig serum, as shown in Figure 2. Compared with the normal control group, the TYR content in the model group was significantly reduced (*P* < 0.05); however, the content of TYR in the CWT groups showed a dose-related increase, especially in the CWT-M and CWT-H groups (*P* < 0.05) (Figure 2(a)), and this was higher than in the BC-treated positive control. MDA, MAO, and AChE contents in serum of the disease model group were significantly increased compared to the normal control group (*P* < 0.05). All doses of CWT significantly reduced the levels of MDA and MAO (*P* < 0.05), but a reduction in AChE was only exhibited in the CWT-H group (*P* < 0.05) (Figures 2(b)–2(d).

### 3.4. The Effect of CWT on TYR, MDA, MAO, and AChE in Skin Tissues

Both mouse and guinea pig skin tissues were used to evaluate the effect of CWT on TYR, MDA, MAO, and AChE levels. Compared with the normal control group, TYR levels in the skin tissue of the disease model group were significantly reduced (*P* < 0.05) (Figures 1(f) and 2(g)), whereas the content of TYR in the BC positive control and CWT groups was noticeably increased (*P* < 0.05) and mainly dose-dependent. The MDA content, higher in the skin tissues of the model group (*P* < 0.05) than the normal control, was decreased after treatment with BC and CWT (*P* < 0.05) (Figures 1(g) and 2(h)). MAO levels were significantly increased in the model group (*P* < 0.05) and slightly decreased in the drug administered groups (Figures 1(h) and 2(i)). The changes in the AChE level in the skin tissues of the two animals were quite different. Negligibly small increases were found in for the disease model mice (*P* < 0.05) (Figures 1(i) and 2(j)), whereas AChE levels were much higher in guinea pigs (*P* < 0.05) (Figure 2(j)). In CWT and BC administered groups, AChE levels were lowered, but the difference was insignificant (*P* < 0.05).

### 3.5. The Effect of CWT on the Expression of TYR, TRP-1, and TRP-2 Protein in Mouse Skin

**Figure 3:**
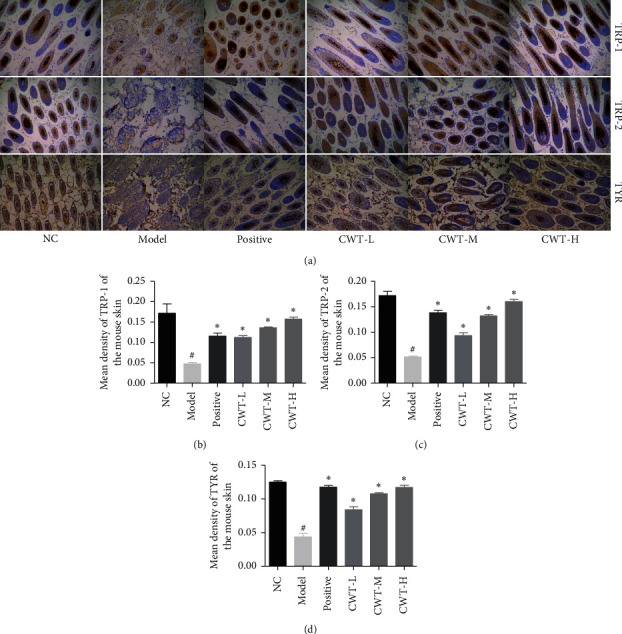
Immunohistochemical staining of shaved mouse skin 200x. (a) Expression of TYR, TRP-1, and TRP-2. (b) Mean density of TRP-1. (c) Mean density of TRP-2. (d) Mean density of TYR. Values are expressed as the mean ± SD of five separate experiments. ^#^#*P* < 0.05 vs. the normal control group. ^∗^*∗P* < 0.05 vs. the model group. NC, blank group; positive, positive control.

The mean density of TYR, TRP-1, and TRP-2 levels in the skin tissue of the model group was significantly reduced (*P* < 0.05) compared to the normal control. In the BC and CWT groups, these were greatly increased (*P* < 0.05), and the effects of CWT were dose-dependent (Figures 3(a)–3(d)).

### 3.6. The Effect of CWT on the Skin and Hair Color of Guinea Pig

**Figure 4:**
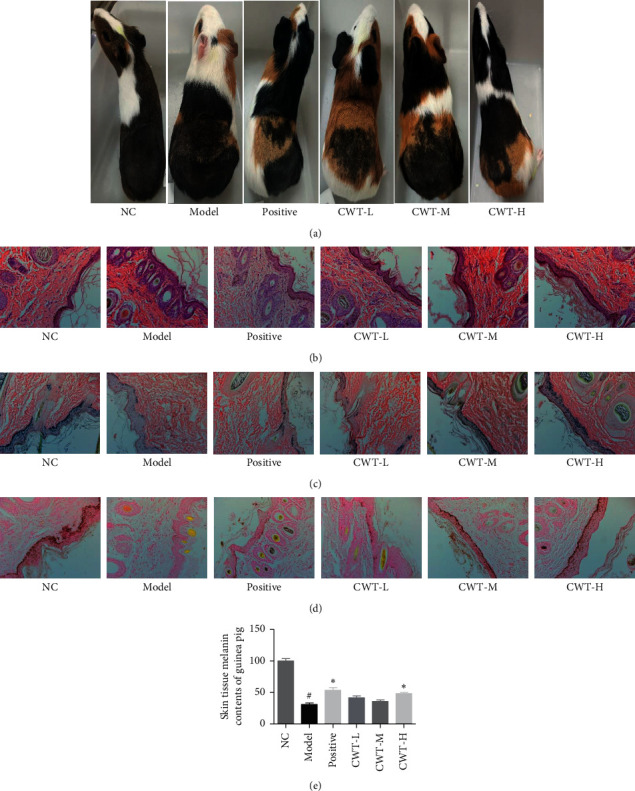
Effect of CWT on guinea pig skin with vitiligo induced by hydrogen peroxide. (a) Effect on hair and skin color. (b) Morphological analysis of skin tissue (H&E staining). (c) Melanin content (Lillie staining). (d) L-DOPA positive cells in the basal layer of guinea pig epidermis—the L-DOPA oxidation method. (e) Distribution of melanin in the epidermis. ^#^#*P* < 0.05 vs. the normal control group, ^∗^*∗P* < 0.05 vs. the model group. NC, blank group; positive, positive control.

In the disease model group, the skin and hair color of the depilated area became lighter compared to the normal control. In the CWT and BC treated groups, the hair color gradually recovered to nearly normal levels (Figure 4(a)).

### 3.7. The Effect of CWT on the Morphology of the Guinea Pig Skin Tissues

Microscopic observation showed that the epidermis of the disease model group was thickened, hyperkeratosis was obvious, and mild inflammatory foci and eosinophils were seen. The pigment content in the spinous and basal layers of the skin was decreased or completely lost. Compared with the untreated group, slight hyperkeratosis was seen in the epidermis of other groups, and lymphocytes and eosinophils were sporadically distributed in the dermis of the model group. (Figure 4(b)).

### 3.8. The Effect of CWT on Melanin Content in the Guinea Pig Skin Epidermis

The experimental results showed that the melanin particles in the skin epidermis of the blank group were more evenly distributed, and both the acanthocyte and the basal cell layer contained melanin particles, which were darker in color. However, in the model group, only a small amount of melanin particles in the basal layer were discovered, and their color was lighter. In the BC group, more melanin particles were seen in the basal cell and spinous cell layers. With each dose group of CWT, more melanin particles, darker in color, were seen in the basal cell layer, as well as in the acanthocyte layer. Moreover, the effect of CWT-H was greater than the BC group (Figures 4(a)–4(e)).

### 3.9. The Effect of CWT on the Distribution of L-DOPA Positive Cells in Guinea Pigs

The results of staining with the L-DOPA oxidation method showed that the basal L-DOPA positive cells of the guinea pig skin epidermis of the untreated group were evenly distributed, and the color was darker (Figure 4(d)). The basal L-DOPA positive cells in the disease model group were considerably reduced, and the color was lighter. The BC positive control showed an increase in distribution of L-DOPA positive cells in the basal layer, and in the CWT-M and CWT-H dose groups, the effect was greater and dose-dependent.

### 3.10. The Effect of CWT on IL-6, INF-*γ* in Blood and *β*-EP, cAMP in the Skin of Guinea Pig

Compared with the untreated group, the contents of IL-6 and INF-*γ* in blood and *β*-EP contents in skin tissue of the disease model group were significantly increased (*P* < 0.05) (Figures 2(e) and 2(f)), while cAMP contents were reduced (*P* < 0.05) (Figure 2(k)). Levels of IL-6 and INF-*γ* in the blood of the CWT-H group was noticeably decreased compared to the disease model group (*P* < 0.05), and the *β*-EP content lowered in all doses of CWT and BC groups (*P* < 0.05) (Figure 2(l)). cAMP contents were raised in the BC and CWT-H groups (*P* < 0.05) (Figure 2(k)).

### 3.11. Effects on Mushroom Tyrosinase Activity

**Figure 5:**
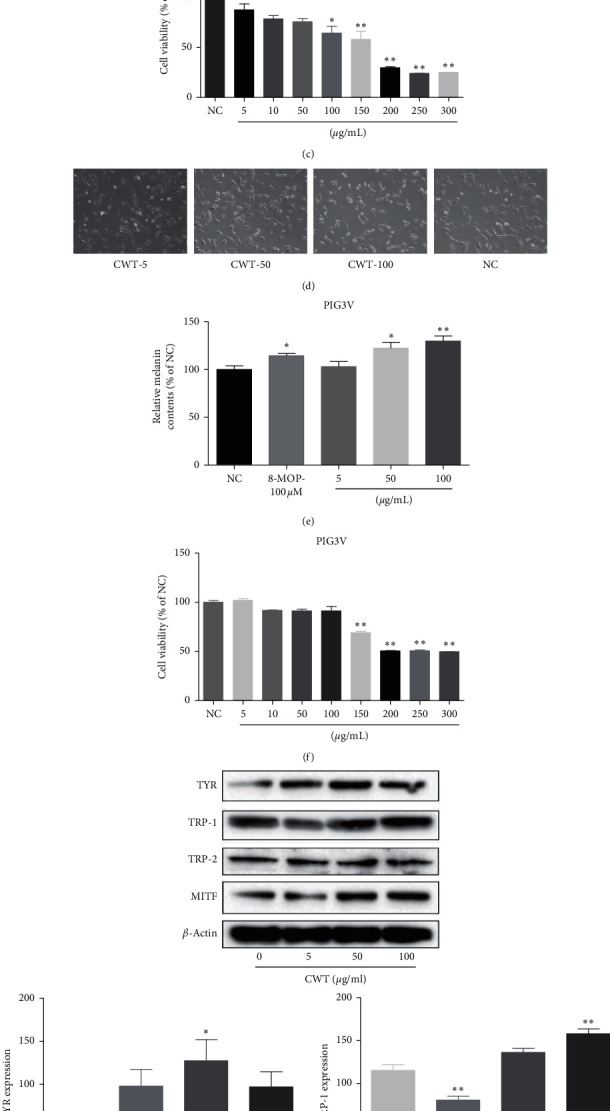
(a) Cell morphology of B16 cells at magnification ×200. (b) Melanin content in B16 cells. (c) B16 cell viability measured by MTT assay. (d) Cell morphology of PIG3V cells at magnification ×200. (e) Melanin content in PIG3V cells. (f) PIG3V cell viability measured by MTT assay. (g) Western blot assays of TYR, TRP-1, TRP-2, and MITF expression levels. Values are expressed as the mean ± SD of three separate experiments. ^∗^*∗P* < 0.05 and ^∗∗^*∗∗P* < 0.01 compared with untreated cells.

The effect of CWT on mushroom tyrosinase activity was investigated in vitro. As the results show, CWT at three different concentrations increased the tyrosinase activity in a dose-dependent manner (Figure 5).

### 3.12. Cytotoxicity of CWT in B16 Melanoma and PIG3V Cells

The effect of CWT on the viability of B16 melanoma cells and PIG3V was examined using the MTT assay. The cells were treated with concentrations of CWT of 5, 10, 50, 100, 150, 200, 250, and 300 *μ*g/mL. 48 h treatment of CWT caused mild cytotoxicity at 100 *μ*g/mL in B16 cells and 150 *μ*g/mL in PIG3V cells, but not at lower dosages (Figures 5(c) and 5(f)), so concentrations between 5 *μ*g/mL and 100 *μ*g/mL were chosen to determine effects on tyrosinase activity and melanin synthesis. CWT increased melanin levels in a dose-dependent manner in both B16 cells and PIG3V cells.

### 3.13. The Effect of CWT on Melanin Synthesis in B16 Cells and PIG3V Cells

To exclude the possibility that a rise in melanin content may be induced by cell-proliferating effects of CWT, the absorbance of the same number of cells across CWT concentrations (5–100 *μ*g/mL) was measured. Melanin levels increased in a dose-dependent manner by CWT treatment in both B16 cells and PIG3V cells (Figures 5(b) and 5(e)). 50 *μ*g/mL was chosen as an effective concentration for further experiments.

### 3.14. The Effect of CWT on MITF and TYR Protein Expression in B16 Cells

Because CWT increased melanin synthesis, we explored whether this was due to expression of MITF, which plays a critical role in TYR gene expression and melanogenesis [8]. The expression of melanogenic genes was explored by Western blot analysis, which indicated that CWT significantly increased protein expression of melanogenic genes TYR, TRP 1, TRP 2, and transcription factor MITF in a nearly dose-dependent manner (Figure 5(g)). Transcriptional levels of MITF, TYR, TRP 1, and TRP 2 were significantly increased in B16 cells in the presence of CWT in a dose-dependent manner.

### 3.15. The Effects of CWT on the PKA Signaling Pathway

**Figure 6:**
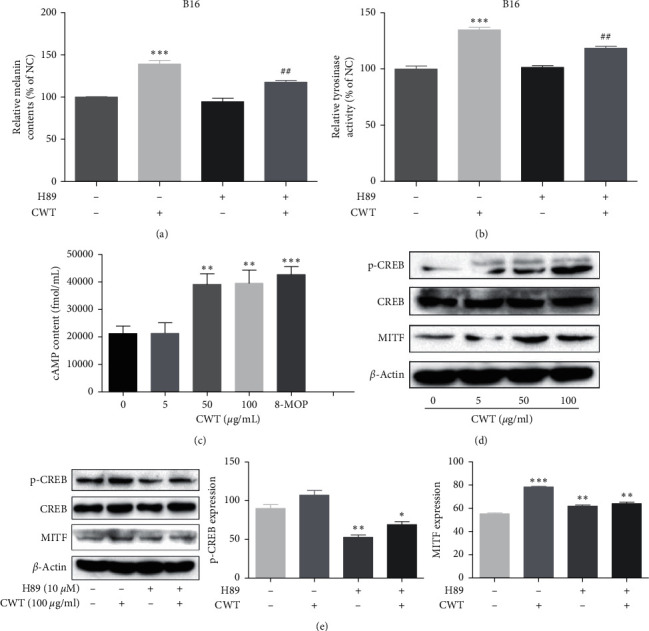
Effects of CWT extract on the PKA signal pathway. (a) The measurement of melanin content. (b) Measurement of TYR activity. (c) cAMP content was measured by a cAMP-ELISA kit. (d) p-CREB, MITF, and CREB measured by Western blot analysis. (e) p-CREB CREB and MITF expression levels proincubated with the inhibitor measured by Western blot analysis. ^∗∗^*∗∗P* < 0.01 and ^∗∗∗^*∗∗∗P* < 0.001 vs. the untreated control group. ^##^##*P* < 0.01 vs. the single treatment group.

The PKA signaling pathway is involved in melanogenesis and promoted cellular cAMP levels to activate PKA [21]. This pathway is an activator for CREB and leads to the upregulation of MITF transcription [22]. To clarify whether the effects of CWT on melanogenesis were also mediated via the PKA signaling pathway, experiments using the PKA inhibitor H89 were conducted, including melanin content measurement, MITF expression, and TYR activity. The effects of CWT on melanogenesis were inhibited after preincubation with H89 (Figures 6(c) and 6(d)). MITF expression was also decreased with H89 (10 *μ*M) and CWT extract (100 *μ*g/mL) (Figure 6(e)). cAMP content (Figure 6(c)) and phosphorylation of CREB were significantly increased in response to CWT (Figures 6(d) and 6(e)) in a dose-dependent manner.

## 4. Discussion

Vitiligo is a widespread and distressing autoimmune skin disease for which there is no proven conventional cure. The pathogenesis of vitiligo and its causes are complex and unclear. The formula “Kursi Karwiya,” or caraway tablet (CWT) has been used as a treatment for vitiligo for decades in Xinjiang, China [11]. We have examined the effects of CWT in models of vitiligo in vitro and in vivo and on melanogenesis in B16 cells and compared them with baidianfeng capsules (BC, a commonly used medicine for vitiligo in China).

Cytotoxicity must be assessed when evaluating the mechanisms and safety of drugs, and the effects of CWT in B16 cells were also examined. No cytotoxic effects at concentrations of 1–50 *µ*g/ml were observed, and in contrast, CWT increased B16 cell proliferation. Melanin content correlates with the activity of tyrosinase and its protein levels [23], so the effect of CWT on tyrosinase activity and expression was further explored. CWT significantly enhanced both tyrosinase activity and melanin synthesis in a concentration-dependent manner (Figures 3(a) and 3(b)), suggesting that it upregulated tyrosinase activity and improved cellular melanin production in B16 cells.

The effect of CWT on melanogenesis in B16 cell lines was further investigated to clarify the underlying molecular mechanisms. In our study, MITF and TYR were increased by CWT, and the degrees were gradually higher to the dose escalation. Intracellular cAMP was increased in a dose-dependent manner, which activates the PKA signal pathway, and the latter activates CREB [22]. Being the main transcription regulator of TYR and upregulated by CREB activation, MITF performs a key function in melanogenesis [24–26]. These results demonstrated that CWT regulated melanogenesis by the PKA signal pathway.

TRP-1 and TRP-2 are transmembrane proteins spanning melanosomal membranes which contribute to modulating TYR activity [27]. CWT increased the expression of TRP-1 and TRP-2 in B16 cells. Melanin-associated signaling pathways are involved in melanogenesis via MITF [28]. The ERK MAPK pathway also participates in melanogenesis [24], and cAMP upregulation activates MAPK in B16 cells and normal human melanocytes [25]. MAPK and PKA pathways modulate the cAMP signal pathway in melanin synthesis [26]. When intracellular cAMP is stored, it activates PKA which subsequently phosphorylates CREB and activates MITF transcription [27]. Our results indicate that CWT activates the PKA signaling pathway via the accumulation of cAMP content, stimulating CREB, leading to the triggering of MITF transcription. MITF upregulates melanogenic genes, including TYR, TRP 1, and TRP 2.

The experimental animal models, using mice and guinea pigs, were chosen based on previous validation of the methods [29–31]. Hydroquinone was used to induce vitiligo in C57BL mice. The monobenzyl ether of hydroquinone is used clinically in patients with vitiligo to complete depigmentation [32, 33]. Hydrogen peroxide was used in the guinea pig model. Reactive oxygen species (ROS) play a role in the pathogenesis of vitiligo, and the loss of epidermal melanocytes may be a result of oxidative stress [34, 35]. Abnormal elevation of epidermal hydrogen peroxide (H_2_O_2_) is seen in lesional and nonlesional skin of vitiligo [36, 37].

Baidianfeng capsule (BC) or “Vitiligo Capsule” is an over-the-counter (OTC) drug, recorded in Chinese Pharmacopoeia (Volume 1, 2020 Edition) and widely used for the treatment of vitiligo [38, 39]. This formula was used as the positive control for the evaluation of CWT. The total content of psoralens in BC is ∼1.85 mg/g [40] and in CWT was ∼3.75 mg/g; however, the dose of BC used, at 720 mg/kg in mice and 270 mg/kg in guinea pig, contained higher concentrations of psoralens than CWT.

Psoralen and isopsoralen act by stimulation of melanin content and tyrosinase activity in melanocytes [41] and by antioxidant activity [42]. Decrease of oxidative stress in vitiligo downregulates key cytokines, blocking melanocyte death and shielding perilesional keratinocytes from damage [43].

CWT-H increased TYR content in blood and skin tissue, as did BC, in the mouse model. MDA in blood and MAO in skin tissue were decreased by CWT and BC, while CWT-H was more active in increasing AChE levels in skin tissue than BC. Immunohistochemical staining was used to identify levels and locations of protein in tissues or organs [44, 45]. The expression of TYR, TRP-1, and TRP-2 in mouse skin in the BC group was significantly upregulated than the model group, and the CWT groups also showed a dose-dependent increase.

Levels of interleukin-6 (IL-6), a proinflammatory cytokine and key factor in the pathogenesis of autoimmune diseases, have been reported to be elevated in vitiligo lesions [46]. Higher IFN-*γ* and IL-6 levels have been observed in the blood and skin of vitiligo patients [47–50] and are indexes for vitiligo diagnosis. [46, 51]. CWT showed a more potent effect than BC in the guinea pig model, unlike in the mouse model. IFN-*γ*, IL-6, *β*-EP, and cAMP contents reverted to the levels of the untreated control group after treatment with CWT, but no significant changes in IFN-*γ* and IL-6 contents were seen with BC. The increase in TYR activity in skin tissue and the decrease in MDA in blood were also significantly dose-dependent for CWT; however, no obvious changes were found in the BC group.

Overall, CWT displayed stronger effects than BC in reverting parameters which were unbalanced in the vitiligo model to near normal levels.

## 5. Conclusion

CWT was able to promote tyrosinase activity and melanin synthesis in B16 cells via activation of the p38 MAPK and PKA signaling pathways. Administration of CWT attenuated the detrimental changes induced by hydroquinone or hydrogen peroxide in two animal models of vitiligo. Our results show that this is at least in part to stimulation of pigmentation and reestablishment of redox balance, blocking melanocyte cell death from damage. CWT exhibited an equivalent effect to BC in the mouse model, and a superior effect in the guinea pig model, despite concentrations of psoralens being lower in the administered dose of CWT than BC. CWT may therefore be a potential medicine for the treatment of vitiligo, but further studies are needed to determine active constituents and clinical applications.

## Figures and Tables

**Table 1:** Dosage regime for treatment of C57BL/6 mice.

Group	Bodyweight, age	Animals per group	Application	Dose of CWT (mg/kg)	Dose of BC (mg/kg)	Dorsal shaved area (mm^2^)
Blank, untreated	20 ± 2 g, 5-6 weeks	12	Distilled water	—	—	20 × 20
Disease model		12	Hydroquinone (5%)	—	—	20 × 20
BC positive control		12	Hydroquinone (5%)	—	720	20 × 20
CWT-L		12	Hydroquinone (5%)	100	—	20 × 20
CWT-M		12	Hydroquinone (5%)	200	—	20 × 20
CWT-H		12	Hydroquinone (5%)	400	—	20 × 20

**Table 2:** Dosage regime for treatment of guinea pigs.

Group	Bodyweight, age	Animals per group	Application	Dose of CWT (mg/kg)	Dose of BC (mg/kg)	Dorsal shaved area (mm^2^)
Blank, untreated	250 ± 20 g, 5–7 weeks	16	Distilled water	—	—	40 × 40
Disease model		16	Hydrogen peroxide (5%)	—	—	40 × 40
BC positive control		16	Hydrogen peroxide (5%)	—	270	40 × 40
CWT-L		16	Hydrogen peroxide (5%)	40	—	40 × 40
CWT-M		16	Hydrogen peroxide (5%)	80	—	40 × 40
CWT-H		16	Hydrogen peroxide (5%)	160	—	40 × 40

**Table 3:** The effect of CWT on the number of melanin-containing hair follicles in mouse skin ($\bar{x}\pm s$).

Group	Samples	Melanin-containing hair follicles
NC	12	45.33 ± 3.26^#^
Model	12	15.47 ± 3.89^*∗*^
Positive	12	24.37 ± 2.54^*∗*^^#^
CWT-L	12	20.80 ± 4.62^∗^*∗*
CWT-M	12	42.33 ± 2.86^#^
CWT-H	12	47.03 ± 2.18^#^

*Note*. Compared with the NC, ^∗^*∗P* < 0.05; compared with the disease model group, ^#^#*P* < 0.05. NC, blank control group; positive, positive control.

## Data Availability

The data generated or analyzed during this study are included within this article.

## Supplementary Materials

Supplementary Materials Supplementary material includes a schematic graph showing the design and the core findings of the study (Figure S1) and the protocol for preparation of caraway tablet based on previous methods (Patent No. CN 107569532 A) which can be found in the text 1 or at https://www.drugfuture.com/cnpat/cn-patent.asp by entering the patent number.
