# Supplementary material for: Effects of a Traditional Caraway Formulation on Experimental Models of Vitiligo and Mechanisms of Melanogenesis
Source: Evid Based Complement Alternat Med. 2021 Apr 19;2021:6675657. doi: 10.1155/2021/6675657 (PMC8075664; doi:10.1155/2021/6675657)
Supplement: Supplementary Materials — Supplementary material includes a schematic graph showing the design and the core findings of the study (Figure S1) and the protocol for preparation of caraway tablet based on previous methods (Patent No. CN 107569532 A) which can be found in the text 1 or at https://www.drugfuture.com/cnpat/cn-patent.asp by entering the patent number. [file 6675657.f1.docx]

**Text 1. Protocol for Preparation of Caraway Tablet**

The formula contains caraway, *Carum carvi L*, as the main constituent with *Psoralea corylifolia L.*, *Tribulus terrestris L.*, *Trachyspermum ammi (L.) Sprague* and *Operculina turpethum L.*, in the ratio 2:1:1:1:1 (total 1002g). Caraway seed was extracted by steam distillation for 5 hours, and the aqueous macerate filtered and collected for later use. The volatile oil was mixed with equal quantities of ethanol, clathrated with β-cyclodextrin (50°C, 2 hours), refrigerated (4°C) for 12 hours, filtered, dried at 40°C, and sieved. *Psoralea corylifolia, Tribulus terrestris, Trachyspermum ammi* and *Operculina turpethum* were mixed, and macerated with 75% ethanol for 12 hours before reflux extraction. The extract was filtered and concentrated, and combined with the caraway aqueous solution described above, concentrated under reduced pressure to a thick paste and dried under vacuum, crushed, and sieved. This was mixed with the clathrate compound and made into a tablet using lactose and substituted hydroxypropyl cellulose with 1% magnesium stearate and compressed into 1000 tablets (0.5g/tablet).


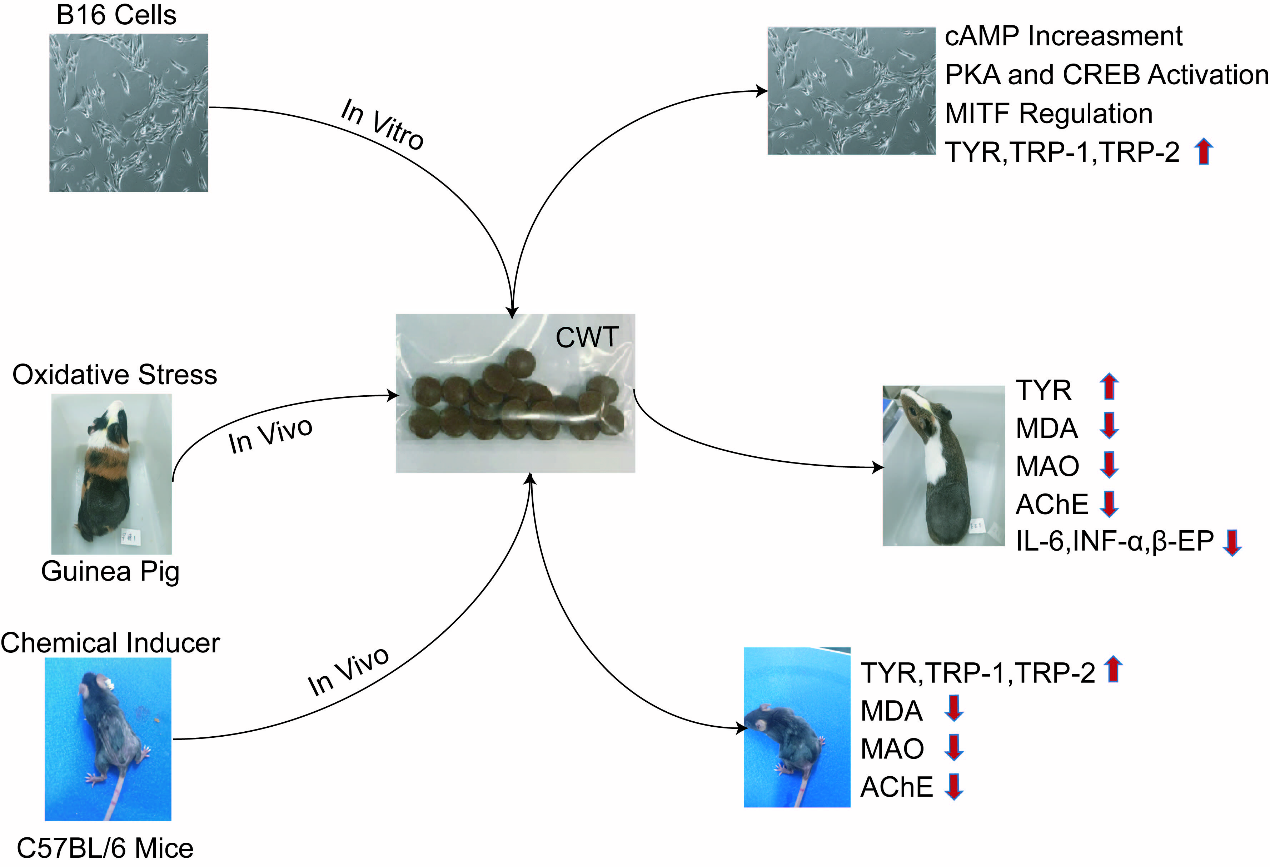


**Figure S 1** A schematic graph showing the design and the core findings of the study
